# Residual inflammation in the cerebrospinal fluid after short- and long-term natalizumab treatment in relapsing-remitting multiple sclerosis

**DOI:** 10.3389/fimmu.2026.1817671

**Published:** 2026-06-05

**Authors:** Sophie Buhelt, Malene Bredahl Hansen, Helle Bach Søndergaard, Sahla El Mahdaoui, Marie Mathilde Hansen, Mie Reith Mahler, Jeppe Romme Christensen, Finn Sellebjerg

**Affiliations:** Danish Multiple Sclerosis Center, Department of Neurology, Copenhagen University Hospital – Rigshospitalet, Glostrup, Denmark

**Keywords:** cerebrospinal fluid, IgG index, natalizumab, oligoclonal band (OCB), relapsing-remitting multiple sclerosis, residual inflammation, soluble B cell maturation antigen (sBCMA), soluble CD27 (sCD27)

## Abstract

**Background and objectives:**

Natalizumab (NTZ) is a high-efficacy therapy for relapsing-remitting multiple sclerosis (RRMS) that blocks peripheral immune cell migration into the central nervous system. While short-term NTZ treatment reduces cerebrospinal fluid (CSF) inflammation, the extent of residual intrathecal inflammation after long-term NTZ treatment and its association with oligoclonal band (OCB) status remains unclear. In this study, we examined associations between NTZ treatment duration, OCB status and CSF inflammatory and tissue biomarkers as well as presence of residual intrathecal inflammation after more than five years of NTZ treatment.

**Methods:**

In this cross-sectional observational study, fifteen inflammatory and tissue biomarkers were reliably measured in CSF using validated immunoassays or extracted from clinical records of 88 NTZ-treated RRMS and 104 untreated RRMS patients as well as 94 controls.

**Results:**

Compared to untreated RRMS patients, most CSF biomarkers were significantly lower in NTZ-treated and controls (Bonferroni-adjusted p (adj-p) < 0.05). However, soluble B cell maturation antigen (sBCMA), soluble CD27 (sCD27), chitotriosidase-1 (CHIT1), IgG index and interleukin-10 (IL-10) were higher in NTZ-treated compared to controls (adj-p < 0.05). NTZ treatment duration was inversely associated with sCD27 levels, and a positive OCB status was associated with higher sCD27 and IgG index (adj-p < 0.05). In patients treated with NTZ for more than five years (n = 41), levels of sCD27, IgG index and sBCMA were higher in OCB-positive patients (adj-p < 0.05), but not in OCB-negative, compared to age-matched controls.

**Discussion:**

Natalizumab treatment reduces CSF inflammation biomarkers, but after more than five years of treatment levels of sCD27, sBCMA and IgG index remain significantly above control levels in OCB-positive NTZ-treated patients. This indicates residual intrathecal adaptive immune activation in OCB-positive NTZ-treated RRMS patients, suggesting that compartmentalized adaptive inflammation in RRMS is incompletely suppressed by NTZ.

## 1 Introduction

Multiple sclerosis (MS) is an immune-mediated disease of the central nervous system (CNS), causing demyelination and neuroaxonal injury leading to neurological dysfunction and disability (1, 2). In its relapsing-remitting phase (relapsing-remitting MS, RRMS), migration of immune cells from the periphery into the CNS is an essential step in the formation of new focal demyelinating lesions (3). However, throughout the MS disease course a parallel, partially independent, slow accumulation of T- and B-cells and innate immune cells occurs in the meninges, perivascular spaces and lesions, resulting in chronic inflammation compartmentalized behind the blood-brain barrier and hence incompletely targeted by current MS therapies (1, 3, 4).

Natalizumab (NTZ) was the first broadly used high-efficacy therapy for RRMS and remains one of the most efficacious treatments available (1, 5). As a monoclonal antibody against very late antigen-4, NTZ blocks immune cells from binding to vascular cell adhesion molecule 1 on endothelial cells, preventing their migration from the periphery into the CNS, thereby inhibiting the inflammatory reaction and focal lesion formation (6).

Cerebrospinal fluid (CSF) is ideal for studying intrathecal inflammation, due to its proximity to the affected tissue in MS. In CSF from MS patients, NTZ treatment leads to decreased levels of inflammatory and tissue-damage biomarkers as well as normalized adaptive and innate immune cell counts and frequencies (7–17). After one year of NTZ treatment, CSF levels of the axonal damage biomarker neurofilament light chain (NfL), the chemokine CXCL13 and the pro-inflammatory cytokine interleukin (IL)-12p40 are at control levels (11, 13, 14). However, soluble CD27 (sCD27), the chemokine CXCL10, chitinase-3-like 1 protein and chitotriosidase-1 (CHIT1) remain above control levels, indicating some residual adaptive and innate immune activation in NTZ-treated patients (8, 13).

A characteristic of MS is the increased production of immunoglobulins within the CNS, compared to controls, which is detected as an increased immunoglobulin G (IgG) index and the presence of oligoclonal bands (OCBs) (18). Although a reduction in IgG index is associated with NTZ treatment (19–23), most NTZ-treated patients remain OCB-positive (OCB_Pos_), indicating continuous intrathecal antibody production, which is in accordance with the observation of intrathecal clonal B cell expansion in NTZ-treated patients (22, 24, 25).

Most previous CSF studies of NTZ-treated patients are characterized by small groups of treated individuals and short treatment durations (≤ one year), resulting in both a low power to detect low-grade intrathecal inflammation as well as a limited understanding of the intrathecal inflammation and tissue damage after longer NTZ treatment duration. Finally, in NTZ-treated patients, associations between OCB status and intrathecal inflammation and tissue damage remains unexplored.

In this cross-sectional observational study, we examine CSF biomarkers of inflammation and tissue damage in a large group of NTZ-treated RRMS patients, many of whom had been treated for more than 5 years. We explore (I) if treatment duration and OCB status are associated with inflammation and tissue-damage and (II) if there is residual inflammation after more than 5 years of NTZ treatment, defined as biomarker levels above those in age-matched controls.

## 2 Methods

### 2.1 Participants

All participants were recruited between 2016 and 2024 at the lumbar puncture outpatient clinic at the Danish Multiple Sclerosis Center at Copenhagen University Hospital – Rigshospitalet (DMSC). Participants were RRMS patients or symptomatic controls (SCs) that had been referred for a lumbar puncture either as part of a diagnostic work-up for neurological disease or treatment monitoring in relation to cessation of NTZ treatment. At the time of lumbar puncture, none of the recruited participants had other neurological disease than RRMS, malignant or autoimmune diseases, or were treated with other systemic disease-modifying therapies than NTZ. Except for a subgroup of NTZ-treated patients (n = 13), none of the participants had received systemic corticosteroids within 4 weeks prior to lumbar puncture.

The SCs were defined in accordance with Teunissens et al.’s guidelines for control groups in CSF biomarker studies (26). All SCs were participants referred for lumbar puncture due to subjective neurological symptoms, however, neurological disease was ruled out by a neurologist due to a normal objective neurological examination and normal paraclinical work-up.

All RRMS patients fulfilled the 2017 McDonald criteria of dissemination in time and space (27). Of the 192 recruited RRMS patients, 104 were newly diagnosed and treatment naïve (untreated RRMS) and 88 were treated with NTZ (NTZ-treated RRMS) for at least three months prior to lumbar puncture, corresponding to a minimum of three NTZ infusions, and had been treatment compliant on NTZ. From the electronic patient records, information on disease onset, most recent relapse and expanded disability status scale (EDSS) prior to lumbar puncture was retrieved, as well as first and last NTZ infusion, treatment prior to NTZ initiation and cause of NTZ treatment cessation. Treatment duration for NTZ was calculated as the months between first NTZ infusion and lumbar puncture. Demographic and clinical characteristics for the RRMS patients and SCs can be seen in **Tables 1** and **2**. The study size was determined by the number of eligible RRMS patients and SCs with available CSF samples recruited during the study period.

**Table 1:** Demographic and clinical characteristics of the natalizumab-treated relapsing-remitting multiple sclerosis (NTZ-RRMS) and untreated relapsing-remitting multiple sclerosis (UT-RRMS) patients and symptomatic controls (SC).

Participant characteristics	NTZ-RRMS	UT-RRMS	SC
N	88	104	94
Female, n (%)	64 (72.7%)	66 (63.5%)	66 (70.2%)
Male, n (%)	24 (27.3%)	38 (36.5%)	28 (29.8%)
Median age at sample collection, years (IQR)	46 (37 - 53)	35 (30 - 44)	37 (29 - 46)
Clinical and paraclinical characteristics			
Median disease duration, months (IQR)	155 (85 - 247)	12 (4 - 50)	
Median EDSS (IQR)^a)^	2.5 (2.0 - 3.5)	2.0 (1.0 - 2.5)	
Relapse within last 12 months, n (%)^b)^	14 (15.9%)	94 (90.4%)	
Oligoclonal bands			
*Positive, n (%)*	64 (72.7%)	96 (92.3%)	
*Negative, n (%)*	24 (27.3%)	8 (7.7%)	
Treatment characteristics			
Methylprednisolone within last 4 weeks, n (%)^c)^	13 (14.7%)	0 (0%)	
Median treatment duration, months (IQR)	53 (26 - 102)		
Reason for treatment change			
*JC-virus positive in serum, n (%)*	*77 (87.5%)*		
*Disease activity, n (%)*	*3 (3.4%)*		
*Other or unknown^d)^, n (%)*	*8 (9.1%)*		
Untreated prior to natalizumab treatment, n (%)^e)^	15 (17.0%)		
Treatment prior to natalizumab, n (%)^e)^	66 (75 %)		
*Daclizumab, n (%)*	*1 (1.1%)*		
*Dimethyl fumarate, n (%)*	*6 (6.8%)*		
*Fingolimod, n (%)*	*9 (10.2%)*		
*Glatiramer acetate, n (%)*	*11 (12.5%)*		
*Interferon-β, n (%)*	*29 (33.0%)*		
*Intravenous immunoglobulin, n (%)*	*2 (2.3%)*		
*Mitoxantrone, n (%)*	*1 (1.1%)*		
*Teriflunomide, n (%)*	*7 (8.0%)*		

^a)^ EDSS was unknown for 3 NTZ-treated and 10 untreated RRMS patients.

^b)^ Relapse within 1 year was unknown for 2 untreated RRMS patients.

^c)^ Methylprednisolone treatment within 4 weeks was unknown for 2 patients.

^d)^ Other causes of lumbar puncture was diagnostic validation (n = 1) and neutralizing antibody positivity (n = 1). The cause was unknown for 6 patients.

^e)^ Treatment prior to natalizumab was unknown for 7 patients.

CSF, cerebrospinal fluid; EDSS, Expanded disability status scale; IQR, Interquartile range.

**Table 2:** Demographic and clinical characteristics of the natalizumab-treated relapsing-remitting MS (NTZ-RRMS) patients that have a short-term (< 24 months), medium-term (24–59 months) and long-term (≥ 60 months) treatment duration.

NTZ-RRMS characteristics	Short-term (< 24 months)	Medium-term (24 - 59 months)	Long-term (≥ 60 months)
N	21	26	41
Female, n (%)	15 (71.4%)	20 (76.9%)	29 (70.7 %)
Male, n (%)	6 (28.6%)	6 (23.1 %)	12 (29.3 %)
Median age at sample collection, years (IQR)	35 (32 - 44)	43 (37 - 49)	51 (45 - 55)
Clinical and paraclinical characteristics			
Median disease duration, months (IQR)	92 (45 - 176)	87 (60 - 206)	216 (149 - 290)
Median EDSS (IQR)^a)^	2.0 (1.5 - 3.5)	2.0 (2.0 - 3.5)	3.5 (1.75 - 4.25)
Relapse within last 12 months, n (%)^b)^	8 (38.1%)	0 (0.0%)	6 (14.6%)
Oligoclonal bands			
*Positive, n (%)*	18 (85.7%)	18 (69.2%)	28 (68.3%)
*Negative, n (%)*	3 (14.3%)	8 (30.8%)	13 (31.7%)
Treatment characteristics			
Methylprednisolone within last 4 weeks, n (%)	8 (38.1%)	3 (11.5%)	2 (4.9%)
Median treatment duration, m (IQR)	11 (6 - 17)	37 (28 - 51)	110 (82 - 143)
Median time from last natalizumab infusion to lumbar puncture, days (IQR)	39 (30 - 50)	32 (22 - 40)	28 (22 - 42)
Untreated prior to natalizumab, n (%)^c)^	7 (33.3%)	5 (19.2%)	3 (8.1%)

^a)^ EDSS score was missing for 1 patient in the medium-term group and 2 patients in the long-term group.

^b)^ Only 6 patients in the short-term group had been treated with natalizumab for ≥ 15 months at the time of lumbar puncture.

^c)^ Prior treatment was unknown for 3 patients in the medium-term group and 4 patients in the long-term group. Treatment with methylprednisolone within 4 weeks was not taken into account.

CSF, cerebrospinal fluid; EDSS, Expanded disability status scale; IQR, Interquartile range.

### 2.2 Standard protocol approvals, registrations, and patient consents

The study was approved by the ethical committee in the Capital Region of Denmark (protocol no.: H-17005703) and was performed in accordance with the Declaration of Helsinki. At recruitment, all participants signed a written informed consent.

### 2.3 Sample collection and storage

The collection and storage of CSF samples were in accordance with the consensus recommendations for CSF collection and biobanking from the BioMS-eu network for CSF biomarker research (28). In a 15 mL polypropylene tube on ice, 12–14 mL CSF was collected by lumbar puncture. Immediately hereafter, the tube was turned 10 times to ensure an equal mixture of CSF proteins. CSF for routine analysis was transferred into new 15 mL polypropylene tubes and the remaining CSF was immediately centrifuged at 400g for 10 minutes at room temperature to isolate the cell-free CSF supernatant, aliquoted into 500 µL volumes in cryotubes, and stored at -80 °C.

### 2.4 Routine CSF analysis

Routine examination of the CSF-serum albumin quotient (QAlb), CSF cell count and IgG index was performed at the Department of Clinical Biochemistry, Copenhagen University Hospital - Rigshospitalet. Cell count in CSF was analyzed using Sysmex (Sysmex Nordic Aps, Copenhagen, Denmark). Exact CSF cell count was only given for counts ≥ 3 cells × 10^6^/L, and counts < 3 cells × 10^6^/L were truncated to 2 cells × 10^6^/L. CSF cell count ≤ 4 cells × 10^6^/L was interpreted as normal. Based on measurements of IgG and albumin in plasma and CSF on VITROS (QuidelOrtho, CA, USA), the IgG index was calculated as $\frac{P-Albumin\;\times \;CSF-IgG}{CSF-Albumin\;\times \;P-IgG}$. An IgG index ≤ 0.67 was interpreted as normal.

Detection of OCBs and NfL measurements were performed at the Neuroimmunology Laboratory at the DMSC. In CSF and serum, OCBs were detected using isoelectric focusing technique on agarose gels followed by immunofixation, using the Hydrasys 2 instrument and the Hydragel 9 CSF Isofocusing kit (Sebia, France). Depending on the number of bands in CSF compared to serum, OCB status was defined as positive if there were ≥ 2 OCBs and negative if there were zero or one band. NfL in CSF was analyzed on a Biotek microplate reader (Synergy HT; Holm & Halby; Broendby, Denmark) using the CE certified enzyme linked immunosorbent assay (ELISA) from UmanDiagnostics (Umeå, Sweden).

### 2.5 Measurement of CSF biomarkers

In previously unthawed CSF samples with an erythrocyte count < 500 × 10^6^/L, the inflammatory biomarkers soluble B-cell maturation antigen (sBCMA), sCD27, CHIT1, CXCL13, interferon-γ (IFN-γ), IL-8, -10, -12p40, -15, -17A, tumor necrosis factor-α (TNF-α), -β (TNF-β) and vascular endothelial growth factor A (VEGF-A), and the tissue biomarkers glial fibrillary acidic protein (GFAP) and myelin basic protein (MBP) were measured. In accordance with manufacturer’s instructions, the biomarkers were either measured by electrochemiluminescence on a MESO™ QuickPlex SQ 120 (MesoScale Discovery, Rockville, MD, USA), by ELISA on a Biotek microplate reader (Synergy HT; Holm & Halby; Broendby, DK) or by single molecule array on a Quanterix SR-X instrument (Quanterix, Billerica, MA, USA). All calibration curves were run in duplicate and evaluated in accordance with the ICH guideline from 2022 on bioanalytical method validation for ligand binding assays (29). For each biomarker, lower limit of quantification (LLOQ) was the lowest approved calibration standard, and measurements below were raised to the LLOQ. Two control samples, respectively designed to measure in the low and high measuring range, were used to determine the inter-assay coefficient of variation (CV) between plates, expressed as percentage (%CV). Biomarkers were excluded from statistical analysis if they had an inter-assay CV > 20% or if neither of the two control samples were detected. All inflammatory- and tissue-damage biomarkers were measured in duplicates, except for CXCL13. Samples above LLOQ with an intra-assay CV above 25% for measurements between the 1^st^ and 2^nd^ lowest approved calibrator, and above 20% for measurements above the 2^nd^ lowest approved calibrator, were excluded from further analysis. For each biomarker, information on the method, manufacturer, dilution, LLOQ, percentage of samples above LLOQ, and inter- and intra-assay %CV are provided in **Supplementary Table 1**.

### 2.6 Statistics

All statistical analyses were performed using SPSS version 29 (IBM, Armonk, NY, USA). Normal distribution was evaluated by the Shapiro-Wilk test and visual interpretation of histograms and QQ-plots. Non-normal distributed biomarkers were analyzed after log_2_ transformation if parametric statistical tests were used. All comparisons between either NTZ-treated and untreated RRMS or SC were performed using an analysis of covariance (ANCOVA) adjusted for age and sex as these demographic characteristics are associated with MS disease course and the levels of some biomarkers (30–32). For analysis of associations between NTZ treatment duration, OCB status and biomarkers, NTZ-treated RRMS patients were categorized into a short-term group if their treatment duration on NTZ was < 2 years (< 24 months), a medium-term group if their treatment duration on NTZ was ≥ 2 and < 5 years (24–59 months) or a long-term group if their treatment duration was ≥ 5 years (≥ 60 months). Associations between treatment duration groups, OCB status, and inflammation and tissue biomarkers were analyzed using ANCOVA, with age, sex, relapses within 1 year of lumbar puncture (binary), high-dose methylprednisolone within 4 weeks prior to lumbar puncture (binary), and days since last NTZ infusion included as covariates. To account for heteroscedasticity in the ANCOVA models, only p-values from parameter estimates with robust standard errors, calculated using the HC3 method, were reported. Statistical analysis of residual inflammation after long-term NTZ treatment was performed by age-matching SCs to the NTZ-treated in the long-term treatment duration group and hereafter analyzed using a non-parametric Mann-Whitney U or Kruskall-Wallis test. Correlations between biomarkers were analyzed using Spearman rank correlation. All statistical analyses were performed using available case data; no imputation of missing biomarker or clinical values was performed. For the biomarkers, all differences and correlations were adjusted for multiple comparisons using the Bonferroni method and were considered statistically significant only if the Bonferroni-adjusted p-value was < 0.05. All differences with an adjusted p-value < 0.05 are shown in tables or graphs. Differences in demographic and clinical characteristics between the NTZ-treated, untreated RRMS and SC groups, as well as the treatment duration groups, were analyzed by a non-parametric Mann-Whitney or Kruskall-Wallis test, or *χ*^2^ test, depending on the data type. Differences in demographic or clinical characteristics are reported as significant if they had a non-adjusted p-value < 0.05. All graphs were created in GraphPad Prism 10.4.1.

## 3 Results

### 3.1 Differences in biomarkers between symptomatic controls, natalizumab-treated and untreated RRMS patients

**Table 1** presents the demographic and clinical characteristics for the 94 SCs and 192 RRMS patients included in the study. In NTZ-treated RRMS, age was significantly higher compared to untreated RRMS patients (p < 0.001) and SCs (p < 0.001). No differences were observed in the distribution of females and males between the SC, NTZ-treated and untreated RRMS groups. Across the groups, significant differences were observed in all biomarker levels except for GFAP, IL-15 and QAlb when adjusting for age and sex (**Table 3**). The biomarkers IL-17A, TNF-β, TNF-α and VEGF-A were excluded from statistical analyses, as either their inter-assay controls were not detected, or they had an inter-assay CV above 20% (**Supplementary Table 1**).

**Table 3:** Inflammation and tissue biomarkers in symptomatic controls (SC), natalizumab-treated (NTZ-RRMS) and untreated RRMS (UT-RRMS) patients.

Biomarker	Group-level analysis							Post-hoc analysis					
	SC		NTZ-RRMS		UT-RRMS		P-value*	UT-RRMS vs SC		UT vs NTZ-RRMS		NTZ-RRMS vs SC	
	n	Mean_Adjusted_ (95% C.I.)	n	Mean_Adjusted_ (95% C.I.)	n	Mean_Adjusted_ (95% C.I.)		Adjusted fold-change (95% C.I.)	P-value*	Adjusted fold-change (95% C.I.)	P-value*	Adjusted fold-change (95% C.I.)	P-value*
Routine													
QAlb	78	4.1 (3.7 - 4.5)	84	4.2 (3.8 - 4.6)	104	4.3 (4.0 - 4.7)	*ns*						
Cells, CSF (mio/L)	94	2 (2 - 2)	88	2 (2 - 3)	104	6 (6 - 7)	**< 0.001**	2.80 (2.42-3.24)	**< 0.001**	2.65 (2.26-3.10)	**< 0.001**	1.06 (0.96-1.16)	*ns*
IgG index	73	0.44 (0.40 - 0.48)	83	0.58 (0.53 - 0.64)	104	0.87 (0.80 - 0.93)	**< 0.001**	1.95 (1.75-2.18)	**< 0.001**	1.48 (1.30-1.69)	**< 0.001**	1.32 (1.21-1.43)	**< 0.001**
T and B cell activation													
sBCMA (pg/mL)	87	80 (71 - 89)	86	107 (95 - 120)	102	226 (203 - 251)	**< 0.001**	2.83 (2.45-3.28)	**< 0.001**	2.11 (1.75-2.54)	**< 0.001**	1.34 (1.18-1.53)	**< 0.001**
sCD27 (pg/mL)	87	212 (182 - 247)	87	433 (370 - 507)	101	1322 (1145 - 1525)	**< 0.001**	6.22 (5.15-7.51)	**< 0.001**	3.05 (2.36-3.94)	**< 0.001**	2.04 (1.69-2.47)	**< 0.001**
Microglial activation													
CHIT1 (pg/mL)	89	1422 (1212 - 1668)	87	1980 (1675 - 2338)	104	3383 (2914 - 3929)	**< 0.001**	2.38 (1.82-3.10)	**< 0.001**	1.71 (1.31-2.22)	**< 0.001**	1.39 (1.14-1.70)	**0.004**
Chemokines and cytokines													
CXCL13 (pg/mL)	87	1.3 (1.1 - 1.6)	85	1.3 (1.1 - 1.6)	102	12.3 (10.3 - 14.6)	**< 0.001**	9.38 (7.16-12.29)	**< 0.001**	9.43 (7.01-12.70)	**< 0.001**	0.99 (0.82-1.20)	*ns*
IFN-γ (pg/mL)	93	0.71 (0.64 - 0.80)	88	0.83 (0.73 - 0.94)	103	1.33 (1.19 - 1.49)	**< 0.001**	1.86 (1.59-2.18)	**< 0.001**	1.61 (1.33-1.94)	**< 0.001**	1.16 (1.03-1.31)	*ns*
IL-8 (pg/mL)	93	33.9 (31.8 - 36.1)	87	36.9 (34.5 - 39.5)	102	45.3 (42.6 - 48.2)	**< 0.001**	1.34 (1.23-1.45)	**< 0.001**	1.23 (1.11-1.36)	**< 0.001**	1.09 (0.99-1.19)	*ns*
IL-10 (pg/mL)	93	0.19 (0.17 - 0.20)	88	0.20 (0.18 - 0.22)	103	0.24 (0.22 - 0.27)	**0.002**	1.32 (1.15-1.52)	**< 0.001**	1.23 (1.06-1.43)	**0.02**	1.07 (1.02-1.14)	**0.03**
IL-12p40 (pg/mL)	93	2.9 (2.5 - 3.2)	88	2.6 (2.3 - 3.0)	103	10.9 (9.7 - 12.3)	**< 0.001**	3.84 (3.18-4.61)	**< 0.001**	4.20 (3.42-5.15)	**< 0.001**	0.91 (0.81-1.02)	*ns*
IL-15 (pg/mL)	93	2.2 (2.1 - 2.3)	88	2.4 (2.3 - 2.5)	103	2.2 (2.1 - 2.3)	*ns*						
*Tissue biomarkers*													
NfL (pg/mL)	90	271 (233 - 316)	85	312 (266 - 367)	104	778 (675 - 897)	**< 0.001**	2.87 (2.31-3.56)	**< 0.001**	2.49 (1.95-3.18)	**< 0.001**	1.15 (0.96-1.38)	*ns*
GFAP (pg/mL)	91	1.3 (1.2 - 1.4)	88	1.3 (1.2 - 1.4)	98	1.2 (1.1 - 1.3)	*ns*						
MBP (pg/mL)	92	286 (254 -322)	87	301 (265 - 341)	104	470 (420 - 526)	**< 0.001**	1.64 (1.38-1.95)	**< 0.001**	1.56 (1.31-1.86)	**< 0.001**	1.05 (0.93-1.19)	*ns*

*All p-values are Bonferroni-adjusted. Only differences with a p-value < 0.05 are interpreted as significant and shown in bold text.

Data were analyzed using an analysis of covariance (ANCOVA) adjusting for age (log2-transformed) and sex. All biomarkers were log2-transformed for statistical analysis and subsequently the adjusted mean and 95% confidence interval (95% C.I.) were back-transformed and presented in the table.

Measurements below lower limit of quantification (LLOQ) were raised to LLOQ. See **Supplementary Table 1** for information on LLOQ and percentage of samples below LLOQ.

sBCMA, soluble B-cell maturation antigen; sCD27, soluble CD27; CHIT1, Chitotriosidase 1; CXCL13, C-X-C motif chemokine ligand 13; GFAP, glial fibrillary acidic protein; IgG Index, immunoglobulin G index; IFN-γ, interferon-γ; IL, interleukin; MBP, Myelin Basic Protein; NfL, neurofilament light chain; ns, non-significant; QAlb, CSF-serum albumin quotient.

In *post-hoc* analyses of the above significant differences, all biomarkers were significantly higher in untreated RRMS compared to SCs and NTZ-treated RRMS (**Table 3**). Between NTZ-treated RRMS patients and SCs, we observed no significant differences in the levels of the tissue damage biomarkers NfL and MBP or the inflammation biomarkers CSF cells, CXCL13, IFN-γ, IL-8 and IL-12p40. However, in NTZ-treated RRMS patients, levels of sBCMA, sCD27, CHIT1, IgG index and IL-10 were significantly above levels in SCs (**Table 3**).

### 3.2 Associations between natalizumab treatment duration and biomarkers

Prior to NTZ treatment initiation, 17% of patients were untreated and 75% had received other MS therapies (**Table 1**). In the NTZ-treated patients, JC-virus antibodies in serum was the most common cause for cessation of NTZ treatment (87.5%), while disease activity was the cause in 3.4% (**Table 1**). Treatment duration in the NTZ-treated RRMS patients ranged from 3 to 173 months, with a median of 53 months (interquartile range (IQR): 26–102 months). Twenty-one had a short-term (< 2 years), 26 a medium-term (≥ 2, < 5 years) and 41 a long-term (≥ 5 years) treatment duration. In **Table 2**, demographic and clinical characteristics are presented for each treatment duration group. Between the treatment duration groups, there was a significant difference in age (p < 0.001), relapse within one year (p = 0.002) and high-dose methylprednisolone treatment within four weeks prior lumbar puncture (p = 0.003) but not sex, OCB status or days between the last NTZ infusion and lumbar puncture.

In the NTZ-treated RRMS patients, we investigated if treatment duration was associated with the level of the measured biomarkers. Interferon-γ and IL-10 were excluded from this analysis as only 11% and 9%, respectively, of their measurements were above LLOQ (**Supplementary Table 1**). Across the treatment duration groups, we observed a significantly lower level of sCD27 with longer treatment durations but not for the other biomarkers (**Table 4**). In *post-hoc* analysis of sCD27, we observed a significant difference in the level of sCD27 (p = 0.002) between the long-term and short-term group but not between the medium-term group and the two other groups.

**Table 4:** Biomarkers of inflammation and tissue-damage in relapsing-remitting MS patients treated with natalizumab short-term (< 2 years), medium-term (≥ 2, < 5 years) or long-term (≥ 5 years).

Biomarker	Short-term (< 2 years)		Medium-term (≥ 2, < 5 years)		Long-term (≥ 5 years)		P-value*
	n	Mean_Adjusted_ (95% C.I.)	n	Mean_Adjusted_ (95% C.I.)	n	Mean_Adjusted_ (95% C.I.)	
Routine							
QAlb	19	4.8 (3.8 - 6.1)	24	4.7 (3.9 - 5.6)	40	4.4 (3.8 - 5.2)	*ns*
Cells, CSF (mio/L)	21	3 (2 - 3)	26	2 (2 - 2)	39	2 (2 - 2)	*ns*
IgG index	18	0.70 (0.60 - 0.81)	24	0.59 (0.53 - 0.67)	41	0.52 (0.47 - 0.57)	*ns*
T and B cell inflammation							
sBCMA (pg/mL)	21	142 (111 - 183)	24	113 (92 - 139)	41	88 (74 - 104)	*ns*
sCD27 (pg/mL)	21	721 (509 - 1021)	25	449 (338 - 596)	41	320 (253 - 406)	**0.04**
Microglial inflammation							
CHIT1 (pg/mL)	21	2665 (1806 - 3934)	25	1726 (1261 - 2361)	41	1755 (1352 - 2278)	*ns*
Chemokines and cytokines							
CXCL13 (pg/mL)	20	1.1 (0.8 - 1.5)	26	1.4 (1.1 - 1.7)	39	1.2 (1.0 - 1.5)	*ns*
IL-8 (pg/mL)	21	38 (32 - 47)	26	38 (33 - 44)	40	37 (33 - 42)	*ns*
IL-12p40 (pg/mL)	21	2.4 (2.0 - 2.8)	26	2.5 (2.2 - 2.9)	41	2.5 (2.2 - 2.8)	*ns*
IL-15 (pg/mL)	21	2.4 (2.1 - 2.7)	26	2.4 (2.2 - 2.7)	41	2.6 (2.3 - 2.8)	*ns*
Tissue biomarkers							
NfL (pg/mL)	20	387 (289 - 519)	26	335 (267 - 420)	39	308 (252 - 375)	*ns*
GFAP (pg/mL)	21	1.6 (1.3 - 2.0)	26	1.4 (1.2 - 1.7)	41	1.3 (1.1 - 1.5)	*ns*
MBP (pg/mL)	21	352 (285 - 437)	25	305 (257 - 363)	39	306 (265 - 354)	*ns*

*All p-values are Bonferroni-adjusted. Only differences with a p-value < 0.05 are interpreted as significant and shown in bold text.

Data were analyzed using an analysis of covariance (ANCOVA) adjusting for age, sex, relapses within 1 year of year lumbar puncture (yes, no), oligoclonal band status (positive (≥ 2 bands), negative (0/1 band)), high-dose methylprednisolone within 4 weeks of lumbar puncture (yes, no) and days between last natalizumab infusion and lumbar puncture. All biomarkers were log2-transformed for statistical analysis and subsequently the adjusted mean and 95% confidence interval (95% C.I.) were back-transformed and presented in the table.

Measurements below lower limit of quantification (LLOQ) for IL-12p40 and CHIT1 were raised to LLOQ. See **Supplementary Table 1** for information on LLOQ and percentage of samples below LLOQ.

sBCMA, soluble B-cell maturation antigen; sCD27, soluble CD27; CHIT1, Chitotriosidase 1; CXCL13, C-X-C motif chemokine ligand 13; GFAP, glial fibrillary acidic protein; IgG index, immunoglobulin G index; IL, interleukin; MBP, Myelin Basic Protein; NfL, neurofilament light chain; ns, non significant; QAlb, CSF-serum albumin quotient.

### 3.3 Associations between OCB status in natalizumab-treated RRMS patients and biomarkers

In NTZ-treated RRMS patients, 73% were OCB_Pos_, which was a significantly lower proportion than in untreated RRMS patients, where 92% were OCB_Pos_ (p-value < 0.001, **Table 1**). Although not significantly different, 86% of the NTZ-treated in the short-term group were OCB_Pos_ compared to 69% and 68% in the medium-and long-term groups, respectively (**Table 2**). For 49 of the 88 NTZ-treated, OCB status at the time of their RRMS diagnosis was accessible through clinical records. At diagnosis 45 of the 49 patients (92%) were OCB_Pos_. In nine of the 45 OCB_Pos_ patients (20%), OCB status had changed from OCB_Pos_ at diagnosis to OCB-negative (OCB_Neg_) at NTZ treatment cessation.

**Figure 1:**
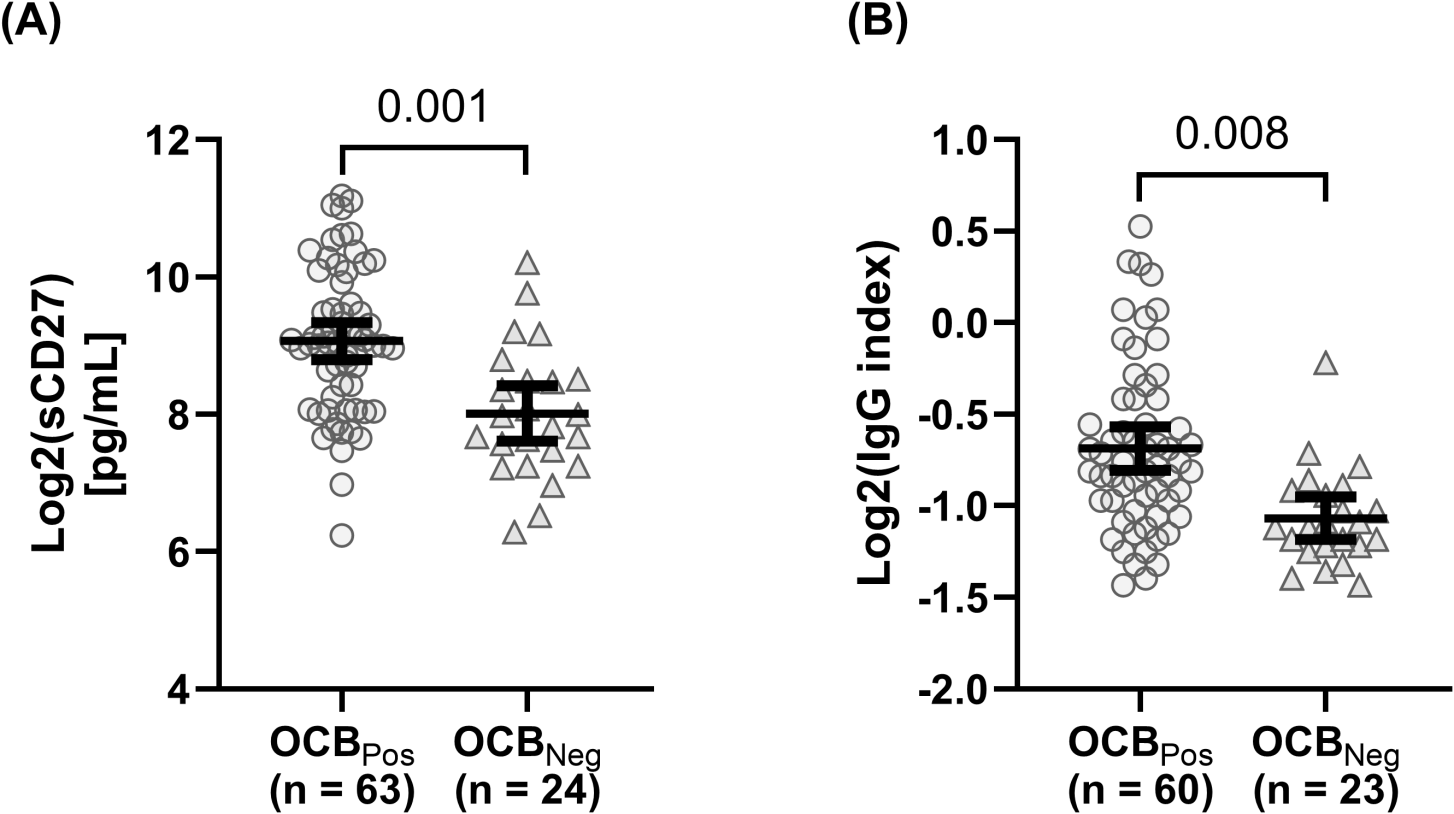
sCD27 and IgG index in oligoclonal band positive (OCB_Pos_) and negative (OCB_Neg_) natalizumab-treated relapsing remitting multiple sclerosis patients. **(A)** Soluble CD27 (sCD27) and **(B)** immunoglobulin G index (IgG index). P-values were calculated using an analysis of covariance and adjusted for age, sex, natalizumab treatment duration (< 2 years, ≥ 2 to < 5 years, ≥ 5 years), methylprednisolone within 4 weeks of lumbar puncture, relapse within 1 year and days between last natalizumab infusion and lumbar puncture. Data are presented log_2_-transformed. Bars indicate the unadjusted mean and 95% confidence interval of the mean. Presented p-values are Bonferroni-adjusted.

Next, in the NTZ-treated RRMS patients we investigated whether OCB status was associated with the levels of inflammation and tissue biomarkers. Using the same ANCOVA as in section 3.2., we observed that OCB status was significantly associated with the level of sCD27 and IgG index (**Figure 1**) but not the levels of the other inflammation or tissue biomarkers (**Supplementary Table 2**). For sCD27, the adjusted mean in OCB_Pos_ was 569 pg/mL (95% CI: 475–681 pg/mL) and 280 pg/mL (95% CI: 209–376 pg/mL) in OCB_Neg_. For IgG index, the adjusted mean in OCB_Pos_ was 0.63 (95% CI: 0.59 – 0.68) and 0.51 (95% CI: 0.45 – 0.58) in OCB_Neg_.

### 3.4 Correlations between tissue and inflammation biomarkers in natalizumab-treated patients

Among inflammation biomarkers in the NTZ-treated RRMS patients, we observed that sCD27 correlated with sBCMA (Spearman’s Rho (r_s_) = 0.86, adjusted p < 0.001) and IgG index (r_s_ = 0.64, adjusted p < 0.001) (**Supplementary Figure 1**). Also, sBCMA correlated with IgG index (r_s_ = 0.48, adjusted p < 0.001), and IL-15 correlated with IL-8 (r_s_ = 0.42, adjusted p = 0.004). In the NTZ-treated RRMS patients, the tissue damage biomarkers NfL and MBP significantly correlated (r_s_ = 0.58, adjusted p < 0.001). Interleukin-15 correlated significantly with MBP (r_s_ = 0.39, adjusted p = 0.01), however, none of the other inflammation biomarkers correlated significantly with tissue biomarkers.

### 3.5 Comparison of sBCMA, sCD27, CHIT1 and IgG index in patients treated with natalizumab ≥ 5 years to age-matched symptomatic controls

Our observation of significantly higher levels of sBCMA, sCD27, CHIT1 and IgG Index in all NTZ-treated RRMS patients compared to all SCs, led us to investigate whether these four biomarkers of inflammation in 41 long-term NTZ-treated RRMS patients were normalized to levels observed in age-matched SCs. The 41 age-matched SCs and 41 long-term NTZ-treated RRMS patients had a similar sex distribution (70.7% females). The median age for the long-term NTZ-treated RRMS patients was 51 years (IQR: 45-55), while for the age-matched SCs it was 47 years (IQR: 43-55). Compared to the age-matched SCs, we observed significantly higher levels of sCD27 (adjusted p = 0.002) and IgG index (adjusted p = 0.01) in the long-term NTZ-treated RRMS patients (data not shown). However, no significant differences were observed in the levels of sBCMA and CHIT1 between the age-matched SCs and the long-term NTZ-treated RRMS patients.

**Figure 2:**
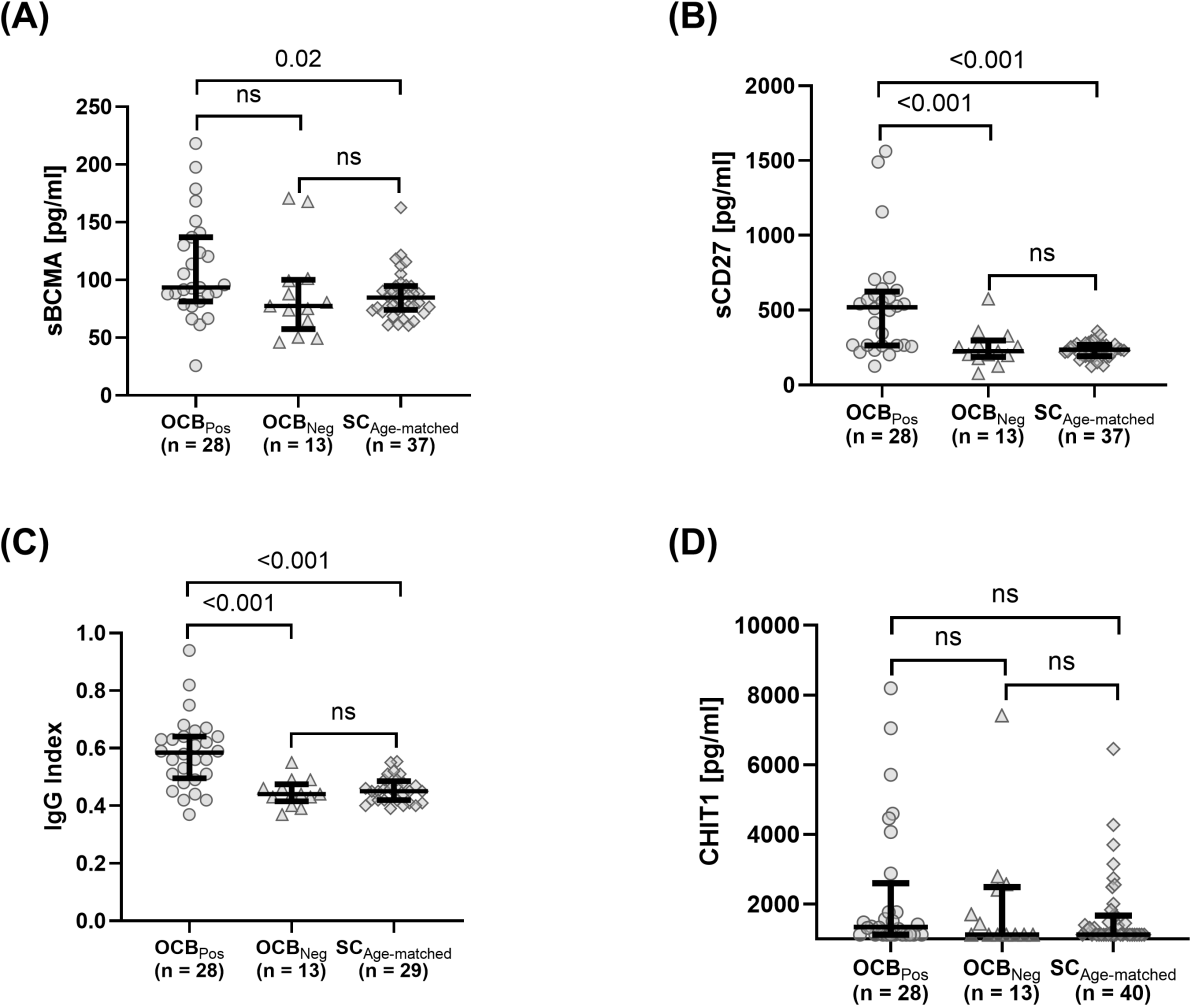
Biomarkers of inflammation in oligoclonal band positive (OCB_Pos_) and OCB negative (OCB_Neg_) relapsing-remitting multiple sclerosis patients treated with natalizumab for more than 5 years and age-matched symptomatic controls (SC_Age-matched_). **(A)** Soluble B-cell maturation antigen (sBCMA), **(B)** soluble CD27 (sCD27), **(C)** immunoglobulin G (IgG) index and **(D)** chitotriosidase 1 (CHIT1). A non-parametric Kruskall-Wallis test was used to calculate p-values. Bars indicate median and interquartile range. Presented p-values are Bonferroni-adjusted.

As OCB status in NTZ-treated RRMS patients was associated with sCD27 and IgG index, we explored if stratification on OCB status affected the above associations. Between the OCB_Pos_ and OCB_Neg_ NTZ-treated RRMS patients there was no significant difference in age, sex, treatment or disease duration, relapse within 1 year or EDSS. In the 28 long-term NTZ-treated positive for OCB there were significantly higher levels of sCD27, sBCMA and IgG index compared to age-matched SCs (**Figure 2**). However, the 13 long-term NTZ-treated negative for OCB had similar levels of sCD27, sBCMA and IgG index as age-matched SCs (**Figure 2**). Oligoclonal band status was not associated with levels of CHIT1 (**Figure 2**).

## 4 Discussion

Natalizumab was the first broadly used high-efficacy therapy for RRMS. As a very late antigen-4 antibody it blocks the recruitment of immune cells from the periphery, thereby inhibiting the formation of new MS lesions. Although many CSF biomarkers of inflammation and tissue-damage are normalized after one year of NTZ treatment, some biomarkers remain increased, suggesting a continued residual inflammation in the CNS (8, 11, 13, 14).

In the present study of a large group of NTZ-treated RRMS patients, many treated for more than 5 years, we investigated whether selected biomarkers in CSF were associated with treatment duration and OCB status and whether there is residual inflammation after more than 5 years of NTZ treatment. All biomarkers studied, except QAlb, IL-15 and GFAP, were lower in SCs and NTZ-treated RRMS compared to untreated RRMS patients, indicating that the biomarkers are MS-associated and responsive to NTZ therapy.

Our study confirms previous findings showing that in NTZ-treated MS patients levels of the axonal damage biomarker NfL, the B-cell chemokine CXCL13 and the cytokine IL-12p40 are comparable to levels observed in controls (11, 13, 14). As a novel finding, we observed that MBP, a myelin damage biomarker, and the chemokine IL-8 were lowered to levels observed in SCs. Due to short NTZ treatment durations, previous studies have been unable to investigate associations with treatment duration. However, we observed no relationship between NTZ treatment duration and the cytokines, chemokines and tissue biomarkers mentioned above, suggesting a swift normalization to control levels after NTZ initiation, as also observed in previous studies (11, 13, 14). This suggests that the primary cellular sources of IL-12p40, IL-8 and CXCL13 either are directly recruited or dependent on cells recruited from the periphery.

As another novel finding, we observed that the levels of sCD27, sBCMA and IgG index in NTZ-treated RRMS patients who are OCB_Pos_, but not OCB_Neg_, were significantly higher than those in age-matched controls, after more than 5 years of NTZ treatment, indicating residual inflammation in long-term NTZ-treated RRMS patients. Both sBCMA and IgG index are believed to originate from antibody-secreting cells while sCD27 presumably originates from T cells and B cells, although the relationship between sCD27 and B cells is unresolved (33–38). As our study observes sCD27 to be associated with OCB status, strongly correlated with sBCMA and moderately with IgG index, it is probable that sCD27 to some extent represents a continuous B cell activation in NTZ-treated RRMS patients. Since NTZ blocks recruitment of immune cells to the intrathecal compartment and cell counts are normalized in NTZ-treated RRMS patients (6, 16, 17), it is most likely that the residual inflammation observed in OCB_Pos_ long-term NTZ-treated RRMS patients is due to continuous activation of tissue-resident B- and T-cells in the CNS. In support of sCD27 representing tissue-resident B- and T-cell activation, a neuropathology study with matched CSF samples observed that sCD27 in CSF correlated with CD3+ and CD20+ cells in meninges and perivascular cuffs and was the best biomarker for predicting lesion activity in post-mortem CNS tissue samples from secondary progressive MS patients (34). Although we do not have neuropathology data, our findings of increased sCD27, sBCMA and IgG index in CSF from OCB_Pos_ long-term NTZ-treated patients are consistent with the idea of ongoing inflammation throughout the MS disease course is caused by a compartmentalization of tissue-resident T- and B- cells, independent of continuous recruitment of peripheral immune cells (3, 4). Furthermore, our results indicate that NTZ insufficiently targets this ongoing inflammation in the majority (68% OCB_Pos_) of RRMS patients even after more than 5 years of treatment, while the OCB_Neg_ patients (32%) had no signs of ongoing inflammation.

Ongoing compartmentalized adaptive inflammation is believed to be involved in progression independent of relapse activity (PIRA), which is insufficiently captured by current biomarkers (1). Interestingly, a recent study found that both kappa free light chains (KFLC), a byproduct of immunoglobulin synthesis, and IgG index were increased in RRMS patients who experienced PIRA, and high levels of KFLC index were found to be an independent predictor of PIRA (39). As it is likely that sCD27 identifies both T- and B-cell inflammation, unlike humoral biomarkers IgG, OCB and KFLC, it could be relevant for future studies to explore if sCD27 is a good biomarker for PIRA. As a diagnostic biomarker, sCD27 has an almost perfect discriminatory power to distinguish inflammatory diseases (including MS) from non-inflammatory diseases, outperforming both IgG index and OCB (33). However, contrary to tissue damage biomarkers, MS-specific changes in inflammation biomarkers in CSF, including sCD27, are not reflected in matched serum samples (32). Therefore, this kind of study would necessitate more frequent CSF collection in the clinical management of MS as well as in clinical trials.

In accordance with a study by Schlüter et al. that observed NTZ treatment duration to be inversely correlated with IgG index (22), we observed a significant inverse correlation between NTZ treatment duration and sCD27. Neither ours nor the study by Schlüter et al. were designed to elucidate if the observed associations are NTZ-dependent or reflect a natural MS disease process. Part of the explanation may be a decrease in T cell infiltrates with higher age and longer disease duration, as observed in a neuropathology study, or that with higher age and/or during NTZ therapy, the intrathecal environment provides less support for the survival of long-lived plasma cells (4, 22, 40, 41).

CHIT1 is a biomarker of microglial activation. Microglia are immune cell native to the CNS and considered important in tissue homeostasis and inflammation (2, 42). In our initial analysis comparing all NTZ-treated RRMS patients with SCs, we observed significantly higher levels of CHIT1 in NTZ-treated. However, in long-term NTZ-treated RRMS patients we observed no significant difference in the level of CHIT1 compared to age-matched SCs. To quantify high levels of CHIT1, we diluted CSF samples 1:20 when analyzing CHIT1, resulting in a high number of CHIT1 measurements below LLOQ especially in SCs (**Supplementary Table 1**). Therefore, the non-significant difference in CHIT1 between SCs and long-term NTZ-treated patients should be interpreted with caution. The median for CHIT1 in long-term NTZ-treated was quantified just above the LLOQ while in age-matched SC it was equal to LLOQ. Therefore, it is possible that low-grade microglial inflammation would have been detectable after 5 years of NTZ treatment if CSF samples for CHIT1 measurements had been diluted less.

Contrary to a previous study analyzing biomarkers in progressive patients after one year of NTZ treatment (43), we observed no correlation between biomarkers of inflammation and tissue damage. The lack of correlation either represents a dissociation between residual inflammation and CNS tissue-damage or indicates that the measured tissue-damage biomarkers insufficiently capture tissue damage when acute inflammatory activity is suppressed by NTZ. Supporting the latter argument, our and previous studies show that NfL and MBP levels are lowered to control levels after NTZ initiation in RRMS patients (13, 14). This suggests that NfL and MBP primarily capture tissue damage associated with the focal bulk invasion of immune cells during relapse activity that is blocked by NTZ, making them undynamic and insufficient to capture the tissue-damage associated with residual inflammation in RRMS patients. Contrary to NfL and MBP, but in accordance with a previous study, we observed no association between NTZ treatment and GFAP, a marker of astrogliosis (14). Neither did we observe an association between GFAP and RRMS. As a biomarker for neuropathological conditions, GFAP in blood outperforms GFAP in CSF (44). This discrepancy remains unresolved, but GFAP seems more unstable in CSF than in blood, as shown by a recent study (44), which may explain the lack of associations.

Our study has some limitations. First, as a cross-sectional study, associations do not necessarily imply causality or direct relationship. Second, we lack baseline or serial CSF sampling of the NTZ-treated group prior to NTZ cessation, which prevents us from estimating the evolution of biomarker levels, as well as estimating the time of OCB seroconversion after the diagnostic lumbar puncture. Regarding our primary aim of estimating residual inflammation in NTZ-treated RRMS patients, we consider this limitation to be minimal since residual inflammation is defined as biomarker levels above those in controls. However, to explore the association between treatment duration and biomarker levels, serial CSF collection would have been a better method, although difficult to perform. Third, few NTZ-treated RRMS patients were treatment naïve prior to the initiation of NTZ. It is likely that treatment-naïve patients would have a lower level of residual inflammation after long-term NTZ treatment due to less accumulation of tissue-resident cells compared to patients who have switched from other therapies. However, this was not possible to explore in our study as only three of the 41 long-term NTZ-treated patients were treatment naïve prior to natalizumab treatment. Fourth, most NTZ-treated patients had received other MS therapies prior to NTZ initiation, and it is possible that these treatments could have influenced the CSF profile in the NTZ-treated patients. Fifth, due to the cross-sectional design, we lacked longitudinal clinical data on disability progression and relapse activity during NTZ therapy; therefore, differences in PIRA in relation to OCB status in the long-term NTZ-treated group could not be evaluated. Such analyses would have provided valuable insights into the clinical relevance of our findings.

In conclusion, we observe that most inflammation biomarker levels in CSF are lower after NTZ treatment. However, after more than 5 years of treatment with NTZ, levels of sBCMA, sCD27 and IgG index remain higher in OCB_Pos_ RRMS patients compared to age-matched controls. These findings are suggestive of residual, compartmentalized inflammation in OCB_Pos_ NTZ-treated RRMS patients indicating that adaptive immune activation in MS may not be completely suppressed by NTZ treatment.

## Data Availability

Data are available in anonymized form and can be shared by request from any qualified investigator. Sharing requires approval of a data transfer agreement by the Danish Data Protection Agency. Requests to access the datasets should be directed to sophie.buhelt.01@regionh.dk.
