# Supplementary material for: Residual inflammation in the cerebrospinal fluid after short- and long-term natalizumab treatment in relapsing-remitting multiple sclerosis
Source: Front Immunol. 2026 Jun 5;17:1817671. doi: 10.3389/fimmu.2026.1817671 (PMC13279040; doi:10.3389/fimmu.2026.1817671)
Supplement: Supplementary file 1 [file SupplementaryFile1.docx]

**Supplementary Figure 1.** Heatmap of correlations between cerebrospinal fluid biomarkers in relapsing-remitting multiple sclerosis patients treated with natalizumab. Spearman’s rank correlation was used. All p-values are Bonferroni-adjusted. *p-value < 0.05, **p-value < 0.005.
